# Temporal stability of quality of life assessments in cancer patients

**DOI:** 10.1038/s41598-021-84681-0

**Published:** 2021-03-04

**Authors:** Andreas Hinz, Thomas Schulte, Jörg Rassler, Markus Zenger, Kristina Geue

**Affiliations:** 1 Department of Medical Psychology and Medical Sociology, University of Leipzig, Philipp-Rosenthal-Str. 55, 04103 Leipzig, Germany; 2 Rehabilitation Clinic Bad Oexen, Bad Oeynhausen, Germany; 3 Department of Urology, St. Elisabeth Hospital, Leipzig, Germany; 4 Faculty of Applied Human Studies, University of Applied Sciences Magdeburg and Stendal, Magdeburg, Germany; 5 Integrated Research and Treatment Center Adiposity Diseases, Leipzig University Medical Center, Leipzig, Germany

**Keywords:** Health care, Quality of life, Oncology

## Abstract

Quality of life (QoL) is an important outcome criterion in cancer research and practice. Multiple studies have been performed to test the short-term temporal stability (1 day–2 weeks) of the European Organization for Research and Treatment of Cancer Quality of Life Questionnaire EORTC QLQ-C30, but its stability over longer periods of time is largely unknown. The EORTC QLQ-C30 was administered at two time points between 3 and 12 months apart in six samples of cancer patients with varying characteristics (N between 298 and 923). Averaged across the six samples, the coefficients of temporal stability (intra-class correlation coefficients ICC) were between 0.31 and 0.59 for the single scales. The 2-item global health/QoL scale showed a mean coefficient of 0.44. When the stability coefficients were calculated separately for males and females and for younger vs. older patients, no systematic gender or age differences were found in the temporal stability of the QoL scales, though the stability was slightly higher in males (vs. females) and in older subgroups (vs. younger subgroups). It is nearly impossible to predict the course a cancer patients’ QoL will take over a several month period. Repeated measurements are necessary to track QoL developments.

## Introduction

Quality of life (QoL) has become an important outcome criterion in medical research. One of the most often used instruments for measuring QoL in cancer patients is the European Organization for Research and Treatment of Cancer Quality of Life Questionnaire EORTC QLQ-C30^1^. Multiple validation studies have proven the psychometric quality of this questionnaire, and several normative studies have been performed^2–4^. However, knowledge about the temporal stability of QoL scores over time periods longer than one month is very limited. Test–retest reliability is considered a quality criterion of questionnaires, and there are multiple studies on test–retest reliability that used time intervals of 1–2 days^5–8^, 3–4 days^9,10^ or 1–2 weeks^11–13^. While these coefficients are relevant for assessing the reliability of the instruments, clinicians are also interested in the question of how well a QoL questionnaire score can predict a patients’ QoL several months later.

There are multiple longitudinal studies that do feature several measurement points spaced months or years apart, but the focus of these studies is generally on measuring mean score changes^14^. In these cases, the researchers aimed to test whether the questionnaire can detect changes resulting from an intervention. However, these mean score changes have nothing in common with the stability or variability of the individual scores within a certain population. Unfortunately, these longitudinal studies generally do not report test–retest correlations, information which would show clinicians how the individual scores might have changed, and to what degree the scores in the different facets of QoL remain stable or change over time, in other words, to what degree the patients’ baseline QoL scores predicts their future QoL. This has consequences for the temporal intervals at which patients’ QoL should be re-addressed.

When cancer patients’ mean QoL self-assessments are compared with those of the general population, a typical finding is that there are strong differences in the functioning and symptom scores between these groups, but that the group differences between the patients and the controls in the assessment of global health/quality of life are small^15^. This can be interpreted as a case of response shift^16^ which is more pronounced in global than in specific dimensions, and leads to the hypothesis that global QoL is more stable than the specific facets of QoL. Concerning age, the detriments younger cancer patients report, as compared with healthy peers, are often more pronounced than the detriments reported by older patients^17^. From this, we derive the hypothesis that temporal stability is higher among older patients than it is in the younger age group. Concerning gender, we hypothesize that the stability of QoL variables is higher among males than among females^18^.

In this paper we compile the results of six studies done among cancer patients. All of these studies used the EORTC QLQ-C30 at least twice at time intervals ranging from 3 to 12 months in length. The specific aims of this paper were (a) to examine the temporal stability of the 15 dimensions of QoL, (b) to test whether global aspects of QoL are more stable than specific aspects, and (c) to examine whether there are age and gender differences in the stability of these variables.

## Methods

### Samples

The data set consists of six different samples with two measurement points each. These six studies have already been analyzed and published with other objectives. We shortly describe the samples and procedures in Table 1 and mention the references concerning further aspects of the studies. We only analyzed the data from respondents who had complete data sets for both t1 and t2. The sample sizes are therefore sometimes lower than those given in the original publications since these publications often only refer to t1. Table 1 gives a summarizing overview of the six samples. While the t1 measurements were performed in the clinic, the t2 questionnaires were completed at home and mailed to the clinic. The format was paper–pencil with the exception of sample 3 (AYAs) where both paper–pencil and online forms were used.

**Table 1:** Characteristics of the six samples.

	REHA	MIXED	AYA	URO-GYN	GYN	BREAST
N	923	897	514	392	298	308
Age (years), mean ± SD	57.1 ± 15.5	60.1 ± 11.9	30.1 ± 6.2	61.2 ± 10.2	61.6 ± 14.2	66.1 ± 9.6
Females (%)	52.0%	40.0%	75.1%	24.7%	100%	100%
Temporal interval, t2-t1 (months)	6 months	6 month	12 months	3 months	3 months	3 months
Global health/QoL, mean ± SD at t1	54.6 ± 19.6	58.0 ± 23.7	66.1 ± 19.3	58.8 ± 26.1	52.1 ± 22.7	63.0 ± 19.3
References	^19^	^20^	^21^	^22,23^	^24^	^25^

#### Sample 1: REHA—mixed cancer patients, rehabilitation clinic

Sample 1 was composed of cancer patients who underwent a rehabilitation program to regain physical fitness (*n* = 923). The most frequent cancer diagnoses were breast cancer (25%), prostate cancer (19%), and cancer of the gastrointestinal tract (18%). Six months after their stay at the rehabilitation clinic (t1) the patients received the t2 questionnaire by mail along with a letter. Further details of the sample have been described elsewhere^19^.

#### Sample 2: MIXED—mixed cancer patients, hospital

This sample included 897 cancer patients who were treated in a large German university hospital. The most frequent cancer localizations were: prostate (19%), breast (11%), rectum (7%), and cervix (6%). The first measurement point was hospital admission, and the second point was six months later^20^.

#### Sample 3: AYA—adolescents and young adults

A sample of 514 AYAs (age 15–39 at diagnosis) was included in this study. The most common tumor diagnoses were breast (27%), non-Hodgkin lymphoma (18%), gynecological tumors (9%), testicular tumor (8%), and hematological cancer (7%). Patients were recruited in 16 German acute care hospitals, four rehabilitation centers, and from two cancer registries. Twelve months after the first measurement point the patients were contacted again^21^.

#### Sample 4: URO-GYN—urological and gynecological cancer patients

This sample was composed of 314 male patients with urologic cancer and 103 female patients with gynecological cancer treated in a German university hospital. In this analysis, we use the data from the first measurement, obtained while the respondents were hospitalized, and the follow-up measurement taken three months later^22,23^.

#### Sample 5: GYN. Gynecologic cancer patients

The participants in this study were 298 patients with gynecological or breast cancer who were recruited in the gynecological clinics of three German hospitals. The first measurement was performed one or two days before hospital discharge, and the second questionnaire was sent to the participants three months thereafter^24^.

#### Sample 6: BREAST—breast cancer survivors

This sample included 308 women who took part in a routine radiologic after-treatment (breast cancer) examination. The mean time since first diagnosis was 7.6 years. The participants were asked to complete the questionnaires immediately after the radiologic examination (t1), and were sent a letter and the t2 questionnaire by mail three months later^25^.

### Ethical approval

The studies were approved by the respective ethics committees. All procedures performed in the studies were in accordance with the 1964 Helsinki declaration and its later amendments of comparable ethical standards.

### Informed consent

Informed consent was obtained from all individual participants included in the studies.

### Instrument

The EORTC QLQ-C30^1^ consists of 30 items which are assigned to five functioning scales, 9 symptom scales (including single symptom items and an item reflecting financial difficulties), and a 2-item global health/QoL scale. All items must be answered with one of four given categories (not at all, a little, quite a bit, very much). The EORTC Quality of Life Group proposed a summarizing score of higher order which is composed of the five functioning scales and eight symptom scales^26^.

### Statistical analysis

The temporal stability was calculated with intraclass correlation coefficients (ICCs). Among the different versions of the ICCs we chose the two-way mixed-effect ANOVA model which is recommended by Qin et al.^27^, and which is model (A,1) in the terminology of McGraw and Wong^28^. ICCs reflect both fluctuations in the ranking of the subjects and mean score changes in the sample. In addition to these ICCs, Pearson correlations were calculated since several studies report test–retest correlations in terms of Persons correlations, and since these coefficients only consider the aspect of the maintenance of the (linear) order of the subjects. Pearson correlations explain the square root of the proportion of the t2 variance explanation. To summarize assessments of the temporal stability across the six samples we calculated the averaged correlations after Fisher’s *z*-transformation. The subsamples according to age and QoL were defined on the basis of the median age and the median global health/QoL score of the sample to get nearly equal sample sizes. Mean score differences between the t1and the t2 measurements were calculated to help interpret the differences between the ICCs and the Pearson correlations. All analyses were performed with SPSS version 24.

## Results

### Temporal stability of the scales

Table 2 presents the ICCs and the Pearson correlations (test–retest correlations) for each of the EORTC QLQ-C30 scales and each sample. The highest stability scores (ICCs) were observed among breast cancer survivors. The right column of Table 2 gives the means of the six coefficients, averaged across the six examinations. They range from 0.31 (nausea/vomiting) to 0.63 (sum score). The mean stability of the 2-item global health/QoL score (ICC = 0.44) was lower than the coefficients of most of the specific scales. When compared with the Pearson correlations, the ICCs were slightly smaller in most cases. Table 3 shows the mean score differences between the t1 and t2 measurements. In the sample BREAST the mean differences were lowest.

**Table 2:** ICCs and Pearson correlations for the total samples.

	REHA		MIXED		AYA		URO-GYN		GYN		BREAST		Mean	
	ICC	r	ICC	r	ICC	r	ICC	r	ICC	r	ICC	r	ICC	r
**Functioning scales**														
Physical	0.64	0.65	0.54	0.57	0.60	0.61	0.45	0.47	0.40	0.40	0.80	0.80	0.59	0.60
Role	0.38	0.43	0.39	0.41	0.45	0.46	0.31	0.34	0.33	0.33	0.69	0.69	0.44	0.45
Emotional	0.63	0.64	0.46	0.47	0.57	0.57	0.51	0.54	0.51	0.51	0.72	0.72	0.57	0.58
Cognitive	0.65	0.65	0.45	0.45	0.56	0.56	0.46	0.46	0.46	0.46	0.73	0.74	0.56	0.56
Social	0.49	0.52	0.42	0.42	0.47	0.48	0.38	0.38	0.45	0.45	0.67	0.68	0.49	0.50
**Global QoL**	**0.39**	**0.44**	**0.43**	**0.43**	**0.45**	**0.45**	**0.40**	**0.45**	**0.28**	**0.29**	**0.63**	**0.63**	**0.44**	**0.45**
Fatigue	0.58	0.61	0.47	0.50	0.52	0.53	0.49	0.50	0.40	0.41	0.74	0.74	0.54	0.56
Nausea/vomiting	0.37	0.38	0.23	0.23	0.26	0.26	0.25	0.25	0.11	0.11	0.60	0.60	0.31	0.32
Pain	0.57	0.59	0.45	0.47	0.52	0.52	0.39	0.40	0.35	0.35	0.72	0.72	0.51	0.52
Dyspnea	0.54	0.55	0.42	0.44	0.44	0.45	0.46	0.46	0.43	0.44	0.77	0.77	0.53	0.53
Insomnia	0.57	0.57	0.43	0.44	0.48	0.48	0.52	0.52	0.40	0.40	0.69	0.69	0.52	0.52
Appetite loss	0.40	0.42	0.30	0.30	0.39	0.39	0.32	0.33	0.23	0.23	0.65	0.66	0.39	0.40
Constipation	0.45	0.46	0.24	0.24	0.43	0.43	0.40	0.40	0.29	0.30	0.70	0.70	0.43	0.44
Diarrhea	0.49	0.49	0.27	0.28	0.34	0.34	0.24	0.24	0.24	0.24	0.56	0.57	0.36	0.37
Financial difficulties	0.66	0.66	0.48	0.49	0.54	0.55	0.60	0.60	0.52	0.53	0.72	0.72	0.59	0.59
**Sum score**	**0.64**	**0.67**	**0.54**	**0.55**	**0.63**	**0.63**	**0.55**	**0.55**	**0.45**	**0.45**	**0.86**	**0.86**	**0.63**	**0.64**

Bold: global or summarizing scores. All of the 96 correlations of the six samples except one (GYN, nausea/vomiting; p < 0.05) are statistically significant with p < 0.001.

**Table 3:** t2-t1 mean score differences.

	REHA	MIXED	AYA	URO-GYN	GYN	BREAST	Mean
**Functioning scales**							
Physical	4.6	− 7.1	3.3	0.1	− 0.1	0.5	0.2
Role	15.3	− 9.1	7.2	− 7.4	− 2.9	− 1.8	0.2
Emotional	5.7	4.5	0.9	9.5	5.0	1.9	4.6
Cognitive	3.8	− 2.6	1.2	2.2	− 2.1	− 1.7	0.1
Social	10.4	− 3.5	7.8	0.2	1.5	2.6	3.2
**Global QoL**	**11.0**	**2.0**	**3.0**	**8.4**	**6.0**	**0.1**	**5.1**
Fatigue	− 9.5	10.3	− 3.5	3.1	5.7	0.2	1.0
Nausea/Vomiting	− 2.3	1.6	− 0.7	− 1.0	1.2	− 0.2	− 0.2
Pain	− 7.6	7.5	− 2.6	− 2.2	3.2	1.2	− 0.1
Dyspnea	− 6.9	8.0	− 4.2	1.6	4.4	0.8	0.6
Insomnia	− 4.4	− 3.4	− 2.0	− 2.1	1.5	− 0.3	− 1.8
Appetite loss	− 6.6	1.0	− 1.4	− 4.2	− 1.2	− 1.0	− 2.2
Constipation	− 5.0	1.4	1.4	2.6	6.9	0.4	1.3
Diarrhea	− 1.5	5.3	− 0.7	− 1.0	0.5	− 1.7	0.1
Financial difficulties	0.2	8.2	− 8.6	3.3	6.0	− 2.6	1.1
**Sum score**	**6.5**	− **3.8**	**2.6**	**0.6**	− **1.5**	**0.2**	**0.8**

Bold: global or summarizing scores.

### Impact of gender, age, and mean QoL on the stability of the QoL scales

Table 4 shows the ICCs of the 2-item global health/QoL scale and the sum score for groups listed by gender, age, and mean QoL scores. This table only presents summarizing QoL measures rather than reporting the results of each individual scale. The right part of Table 4 presents the mean stability coefficients (ICCs). Two samples (GYN and BREAST) included only females, and one sample (AYA) included only adolescents and young adults. We did not calculate age-specific results for the latter sample due to the limited age distribution. Averaged across all samples, the males’ scores were slightly more stable than the females’ scores, and older patients’ scores were slightly more stable than those of younger patients. The single samples’ results were mixed, however. While in the URO-GYN sample, males were much more stable than females, the other samples showed only small and unsystematic differences between males and females. Concerning age, in three of the five samples (MIXED, URO-GYN, GYN), the older subgroups reached higher stability coefficients than the younger groups, while the coefficients were nearly equal in the REHA sample, and in the BREAST sample we observed an opposite trend.

**Table 4:** Temporal stability (ICCs), listed by subgroups.

	REHA		MIXED		AYA		URO-GYN		GYN		BREAST		Mean	
**Gender**	M	F	M	F	M	F	M	F	–	–	–	–	M	F
Global QoL	0.39	0.38	0.43	0.39	0.47	0.43	0.46	0.17	–	–	–	–	0.44	0.35
Sum score	0.64	0.61	0.54	0.50	0.56	0.62	0.57	0.32	–	–	–	–	0.58	0.52
**Age**	Yg	Old	Yg	Old	–	–	Yg	Old	Yg	Old	Yg	Old	Yg	Old
Global QoL	0.38	0.40	0.38	0.48	–	–	0.39	0.41	0.25	0.30	0.67	0.58	0.42	0.44
Sum score	0.63	0.64	0.47	0.60	–	–	0.48	0.62	0.36	0.52	0.86	0.85	0.59	0.69
**QoL**	Low	High	Low	High	Low	High	Low	High	Low	High	Low	High	Low	High
Global QoL	0.10	0.36	0.19	0.14	0.19	0.25	0.12	0.28	0.09	0.24	0.31	0.43	0.17	0.29
Sum score	0.47	0.63	0.45	0.31	0.49	0.55	0.40	0.43	0.45	0.28	0.80	0.82	0.53	0.53

*M* males, *F* females, *Yg* young (≤ median), *Old* (> median), *Low* global health/QoL ≤ median, *High* global health/QoL > median.

In four of the six samples, the stability among the patients with relatively high QoL at t1 was higher than the corresponding scores of the low QoL groups, but in one sample (MIXED) there was an opposite trend, and in one further sample (GYN), the results were mixed, with higher stability of the global health/QoL score and lower stability of the QoL sum score for the high QoL subgroup.

## Discussion

As was to be expected, the coefficients of temporal stability were much smaller than those obtained in studies with time intervals of only a few days between measurement points. In the nine studies featuring time intervals between 1 day and 2 weeks in lengths^5–13^ the stability coefficients of the 2-item global health/QoL scale were between 0.82 and 0.93, with the exception of one study that examined brain tumor patients. The averaged Pearson correlation our study found (*r* = 0.45) for measurement periods between 3 and 12 months long means that only 20% (*r*^2^ = 0.202) of the variance of the t2 measurement can be explained by the t1 scores. The relatively low stability coefficients (as compared with those reported above) in our samples can have two reasons: longer temporal intervals and changes in the health situation of the patients from t1 to t2. Among the six studies, the sample of breast cancer survivors showed the highest stability coefficients in all scales, a result which is obviously due to the absence of treatment and the longer period of time that had elapsed since diagnosis (7.6 years) compared with the other samples. While Pearson correlations only indicate changes in the (linear) rank position of the individuals, ICCs are also sensitive to mean score changes. Therefore, we focused on these ICCs. However, the compilation of ICCs and Pearson correlations in Table 2 shows that in most cases the differences between these two types of coefficients are small in magnitude. Temporal stability as measured with the ICC or with Pearson correlations should not be considered a criterion of psychometric quality of the instrument. This would only be justified if no systematic changes in the patients’ health situation had occurred. This however is obviously not the case. Low temporal stability coefficients do not mean that the questionnaire is unreliable. The stability coefficients provide clinicians with information about the degree of precision with which patients’ future QoL can be predicted on the basis of a baseline measurement.

We had hypothesized that global health/QoL would be more stable than the specific facets of QoL. This was not confirmed. For most of the functioning scales, the stability was higher than that of the global health/QoL scale, and when compared with the symptom scales, the stability of the global health/QoL was in the middle range. This means that, even if there are only slight changes of the global assessment of health and QoL on the group level^29^, there are nevertheless remarkable changes in the individual assessments.

The sum score of the EORTC QLQ-C30 showed the highest stability scores. Since calculating sum scores is relatively new in the research on the EORTC QLQ-C30, there are no studies in the literature which have reported the stability of this sum score. The summarizing assessment of QoL was markedly more stable (averaged ICC = 0.63) than the 2-item global health/QoL scale (ICC = 0.44). This underlines the usefulness of this sum score when a generalized assessment of QoL is intended.

To our knowledge, this is the first study that explicitly examined gender and age differences in temporal stability of QoL. Females generally report higher levels of anxiety, emotional lability and neuroticism than males^18^. However, our results show that this does not necessarily mean that females are inconsistent in their judgments. Four of the samples profiled here included males and females, two of which showed higher stability scores for the males, while the other two showed mixed results. Here one has to take into account that most of the female participants were post-menopausal; the individual changes might be more pronounced among younger women. Concerning age, on average older patients gave somewhat more stable responses than younger patients did, a tendency which was observed in three of the five samples which included age comparisons. Taken together, we detected only inconsistent age and gender tendencies in the stability of QoL assessments. Further research is needed to examine whether it is really necessary to consider age and gender peculiarities of the samples when studying the stability of QoL assessments.

This study has several strengths and limitations. One strength is the relatively large sample size given that longitudinal study samples with more than 300 participants are rare. This gave us the opportunity to divide the samples into subgroups and to address the questions of how age and gender differences impact stability. The comparison of the six samples gives an impression of the generalizability of the results obtained from a single examination. A limitation of this study is the heterogeneity of the six samples in terms of cancer type, treatment protocols, and stages. Nevertheless, even readers who question the justification of averaging the stability values over these heterogeneous studies may still be interested in the results of the six individual examinations. In the cases where low temporal stability was observed it remains unclear to what degree that instability was due to natural fluctuations or to individual differences in disease processes. The relatively low stability coefficients of some of the one-item symptom scales may also be due to low reliability of the measure and to real changes. We chose to average (after *z*-transformation) the correlation coefficients of the studies with different sample sizes. Another option would be to weight the samples according to the sample sizes. Doing this would however have resulted in different weights for the different settings. We could not discuss all relevant aspects of age and gender differences in the fluctuations of QoL responses, we could not present and discuss the age and gender differences of the 15 scales of the EORTC QLQ-C30, and we could not discuss the peculiarities of the six samples in more detail.

Nevertheless, our general conclusion is that measuring QoL in a hospital setting does not provide sufficient information on what QoL the patient may expect some months later. Repeated measurements are necessary to follow individual courses of QoL.

## References

[1] Aaronson NK (1993). The European-Organization-For-Research-And-Treatment-Of-Cancer QLQ-C30: A quality-of-life instrument for use in international clinical trials in oncology. J. Natl. Cancer Inst..

[2] Hinz A, Singer S, Brähler E (2014). European reference values for the quality of life questionnaire EORTC QLQ-C30: Results of a German investigation and a summarizing analysis of six European general population normative studies. Acta Oncol..

[3] Nolte S (2019). General population normative data for the EORTC QLQ-C30 health-related quality of life questionnaire based on 15,386 persons across 13 European countries, Canada and the Unites States. Eur. J. Cancer.

[4] Nolte S (2020). Updated EORTC QLQ-C30 general population norm data for Germany. Eur. J. Cancer.

[5] Liu J (2019). Reliability, validity and responsiveness of the Mandarin (Simplified) Chinese version of the EORTC QLQ-OH45 among cancer patients. Eur. J. Cancer Care.

[6] Cheng J-X (2011). The validation of the standard Chinese version of the European Organization for Research and Treatment of Cancer Quality of Life Core Questionnaire 30 (EORTC QLQ-C30) in pre-operative patients with brain tumor in China. BMC Med. Res. Methodol..

[7] Wan C (2008). Validation of the simplified Chinese version of EORTC QLQ-C30 from the measurements of five types of inpatients with cancer. Ann. Oncol..

[8] Lundy JJ, Coons SJ, Aaronson NK (2015). Test-retest reliability of an interactive voice response (IVR) version of the EORTC QLQ-C30. Patient.

[9] Hjermstad MJ, Fossa SD, Bjordal K, Kaasa S (1995). Test/retest study of the European Organization for Research and Treatment of Cancer core quality-of-life questionnaire. J. Clin. Oncol..

[10] Nejjari C (2014). Translation and validation of European organization for research and treatment of cancer quality of life Questionnaire -C30 into Moroccan version for cancer patients in Morocco. BMC Res. Notes.

[11] Silpakit C (2006). The European Organization for Research and Treatment of Cancer Quality of Life Questionnaire (EORTC QLQ-C30): Validation study of the Thai version. Qual. Life Res..

[12] Chie W-C, Chang K-J, Huang C-S, Kuo W-H (2003). Quality of life of breast cancer patients in Taiwan: Validation of the Taiwan Chinese version of the EORTC QLQ-C30 and EORTC QLQ-BR23. Psycho-Oncol..

[13] Magaji BA, Moy FM, Roslani AC, Law CW, Sagap I (2015). Psychometric validation of the Malaysian Chinese version of the EORTC QLQ-C30 in colorectal cancer patients. Asian Pac. J. Cancer Prevent: APJCP.

[14] Onate-Ocana LF (2009). Validation of the Mexican Spanish version of the EORTC C30 and STO22 questionnaires for the evaluation of health-related quality of life in patients with gastric cancer. Ann. Surg. Oncol..

[15] Arndt V, Merx H, Stegmaier C, Ziegler H, Brenner H (2004). Quality of life in patients with colorectal cancer 1 year after diagnosis compared with the general population: A population-based study. J. Clin. Oncol..

[16] Sprangers MAG, Schwartz CE (1999). Integrating response shift into health-related quality of life research: a theoretical model. Soc. Sci. Med..

[17] Hinz A (2018). Quality of life in cancer patients-a comparison of inpatient, outpatient, and rehabilitation settings. Support Care Cancer.

[18] Daig I, Herschbach P, Lehmann A, Knoll N, Decker O (2009). Gender and age differences in domain-specific life satisfaction and the impact of depressive and anxiety symptoms: A general population survey from Germany. Qual. Life Res..

[19] Friedrich M (2019). Measuring fatigue in cancer patients: A common metric for six fatigue instruments. Qual. Life Res..

[20] Hinz A (2010). Anxiety and depression in cancer patients compared with the general population. Eur. J. Cancer Care.

[21] Leuteritz K (2018). Life satisfaction in young adults with cancer and the role of sociodemographic, medical, and psychosocial factors: Results of a longitudinal study. Cancer.

[22] Zenger M, Brix C, Borowski J, Stolzenburg J, Hinz A (2010). The impact of optimism on anxiety, depression and quality of life in urogenital cancer patients. Psycho-Oncol..

[23] Zenger M, Glaesmer H, Höckel M, Hinz A (2011). Pessimism predicts anxiety, depression and quality of life in female cancer patients. Jpn J. Clin. Oncol..

[24] Thieme M, Einenkel J, Zenger M, Hinz A (2017). Optimism, pessimism and self-efficacy in female cancer patients. Jpn J. Clin. Oncol..

[25] Fuhrmann K, Mehnert A, Geue K, Hinz A (2015). Fatigue in breast cancer patients: psychometric evaluation of the fatigue questionnaire EORTC QLQ-FA13. Breast Cancer (Tokyo).

[26] Giesinger JM (2016). Replication and validation of higher order models demonstrated that a summary score for the EORTC QLQ-C30 is robust. J. Clin. Epidemiol..

[27] Qin S, Nelson L, McLeod L, Eremenco S, Coons SJ (2019). Assessing test-retest reliability of patient-reported outcome measures using intraclass correlation coefficients: Recommendations for selecting and documenting the analytical formula. Qual. Life Res..

[28] McGraw KO, Wong SP (1996). Forming inferences about some intraclass correlations coefficients. Psychol. Meth..

[29] Hinz A (2017). The relationship between global and specific components of quality of life, assessed with the EORTC QLQ-C30 in a sample of 2019 cancer patients. Eur. J. Cancer Care.

